# Therapeutic Effect of Nile Tilapia Type II Collagen on Rigidity in CD8^+^ Cells by Alleviating Inflammation and Rheumatoid Arthritis in Rats by Oral Tolerance

**DOI:** 10.3390/polym14071284

**Published:** 2022-03-22

**Authors:** Chunyu Hou, Na Li, Mengyao Liu, Jingjing Chen, Jeevithan Elango, Saeed Ur Rahman, Bin Bao, Wenhui Wu

**Affiliations:** 1 Department of Marine Biopharmacology, College of Food Science and Technology, Shanghai Ocean University, 999 Hu Cheng Huan Road, Shanghai 201306, China; ytxiaoyu@163.com (C.H.); 18335440803@163.com (N.L.); 13866250427@126.com (M.L.); jingjingchen86@163.com (J.C.); srijeevithan@gmail.com (J.E.); bbao@shou.edu.cn (B.B.); 2 Oral Biology, Institute of Basic Medical Sciences, Khyber Medical University, Peshawar 25000, Pakistan; saeedbio80@gmail.com

**Keywords:** type II collagen, molecular structure, immune tolerance, cytokines

## Abstract

Fibrillins are microfibril-associated macro glycoproteins found in connective tissues and structurally related to latent TGF-β-binding proteins (LTBPs). The special cellular immunity and blocking glycoprotein receptors IIb and IIIa of fibrillins are emerging topics in recent years. In this study, Nile Tilapia type IIcollagen (NTCII) was extracted and purified from the skull cartilages by a pepsin-soluble method. Amino acid analysis indicated that NTCII consisted of 315/1000 glycine residues, 72/1000 hydroxyproline residues and 108/1000 proline residues. SDS-PAGE analysis showed that NTCII was composed of three identical 130 kDa α-chains. The results of glycoprotein/carbohydrate assay indicated that the total polysaccharide content of NTCII was 5.6–19.0%. The IR spectrum of NTCII displayed five characteristic peaks of amide I, II, III, A, B. NTCII at 10–100 μg/mL concentration downregulated the content of cytokines in the presence or absence of LPS, especially the secretion of cytokines IL-6, IL-1β and TNF-α. Interestingly, NTCII promoted the secretion of Fas/Apo-1 compared to the control group and 25 μg/mL of NTCII resulted in a higher Fas/Apo-1 secretion level in CD8^+^ T cells. FITC-TCII fluorescence images confirmed that NTCII could bind to the membrane surface of CD8^+^ T cells, leading to the induction of rigidity. NTCII could bind to the membrane surface of CD8^+^ T cells that leads to the induction of rigidity, as evidenced by the FITC-NTCII fluorescence images. The qRT-PCR gene expression analysis of caspase-8 collected with Fas/Apo-1 was upregulated significantly in the 1 and 50 μg/mL NTCII-treated groups compared with the control group. Overall, the results conclude that the rigidity did not lead to an increase in inflammatory factors in CD8^+^ T cells treated with NTCII. The oral administration of NTCII 3 mg/kg dosage caused more prominent repair of damaged ankle cartilage than the 1 mg/kg dosage in Freund’s adjuvant-induced model of arthritis in rats. Therefore, this study disclosed the immunological and anti-arthritic effect of fibrillar collagen, which could be a potential biomaterial for practical applications with lower toxicity.

## 1. Introduction

CD8^+^ T cells can encounter cognate antigens presented by nearly any cell type. Dendritic cells (DCs) can stimulate naive CD8^+^ T cells [1], and all systemically and peripherally expressed antigens can be presented to naive CD8^+^ T cells circulating by the secondary lymphoid tissue. Many activated CD8^+^ T cells can induce DC activation [2]. Peter et al. found that the delivery of a single dose of peptide to mice containing viral glycoprotein (GP)-specific CD8^+^ T cells resulted in T cells priming and memory cell formation, whereas multiple doses resulted in tolerance [3]. In this case, CD8^+^ T cells appeared to occur by deletion. The establishment and maintenance of T cells tolerant to self and non-pathogenic foreign antigens are critical for immune homeostasis. One of the key mechanisms of the immune response or immune tolerance depends on the activity of CD8^+^ T cells. When an immune response occurs, T-cell antigen receptors (TCRs) can induce a series of signal transductions that play a key role in the functional activity of T cells [4].

Oral tolerance is the induction of immunological hypo-responsiveness toward specific antigens or other antigens present at the site through a bystander effect via the secretion of immunosuppressive cytokines [5]. Research showed that lymphocytes from mice tolerized by ovalbumin feeding die rapidly when cultured in vitro after restimulation with antigen in an adjuvant in vivo, and that these changes were reduced in the presence of antigen [6]. Furthermore, oral tolerance has been recently applied as a treatment for human autoimmune diseases. Low doses of β_2_-glycoprotein I probably via induction of CD4^+^ and CD8^+^, suppress T cells. Studies have shown that oral tolerance is enhanced by varying the delivery vehicle or by incorporating immunomodulators such as LPS and cholera toxin B subunit [7].

Due to excellent biocompatibility and oral tolerance, fibrous glycoprotein type II collagen has been used for treatment against autoimmune diseases [8]. It has been reported that oral collagen obtained from cattle, chicken, and sheep have a therapeutic effect on rheumatoid arthritis (RA) [9,10,11]. The influence of chicken type II collagen on the inflammation and immune response of mesenteric lymph node lymphocytes (MLNLs) was investigated in a rat CIA model established by Zhao et al. [11]. Type II collagen-responsive T cells play a crucial role in the progression and duration of rheumatoid synovial inflammation by leading the production of Th1-related cytokines, pro-inflammatory cytokines, and inflammatory chemokines [12]. It has been indicated that type II collagen can promote the expression of apoptosis-regulating genes in human leukemia cells, which is the mechanism of immune tolerance of type II collagen in the blue shark [13]. Type II collagen forms a reticular fiber structure in cartilage, which makes it easier to combine proteoglycan with other components to maintain the balance of matrix components, to ensure that cartilage has excellent elasticity, tensile strength and shock absorption characteristics. The integrity of type II collagen reticular structure ensures many physiological functions of cartilage.The mechanism of glycoprotein induced immune tolerance still needs to be explored.

At present, research into using Nile tilapia type II collagen (NTCII) for the treatment of immune tolerance has mainly focused on investigating cytokine levels in vitro, and the in-depth research on the molecular mechanism of cell signal transduction is unexplored. Based on the species diversity of marine type II collagen [14], this study has focused on exploring the rigidity characteristics of CD8^+^ T cells induced by a fibrous glycoprotein NTCIIin vitro and the oral tolerance of NTCII in a RA model in vivo.

## 2. Materials and Methods

### 2.1. Materials

Farmed Nile tilapia skulls were purchased from Shanghai Fisheries Research Institute, Shanghai, China and stored at −80 °C until processing. Pepsin, Sodium dodecyl disulfate (SDS), Hydroxyproline standards and Ammonium persulphate (AP) (Sigma-Aldrich Ltd., Shanghai, China); Standard protein marker (3.5~245 kDa) (BBI Life Sciences, Shanghai, China); CD8^+^ T cells (Shanghai Zhong Qiao Xin Zhou Biotechnology Co., Ltd., Shanghai, China); RPMI 1640 medium and fetal bovine serum (FBS) (Gibco ThermoFisher Scientific Company, USA); ELISA kit (Nanjing Jiancheng Bioengineering Institute, Jiangsu P, China); Trizoland RT-PCR primers (Shanghai Generay Biotech Co., Ltd, Shanghai, China). Kits for cDNA first-strand synthesis and Talent fluorescence quantitative detection (SYBR Green, Beijing, China); Formaldehyde, ethylenediaminetetraacetic acid (EDTA), neutral formalin, absolute ethanol, section paraffin, xylene, hematoxylin-eosin (HE) and neutral resin (China National Medicines Co., Ltd, Shanghai, China).

### 2.2. Extraction of Collagen

Nile tilapia skull was used for the extraction of collagen as described in our previous methods [1,15]. All extraction procedures of type II collagen were performed at ≤4 °C. Cut the samples into small pieces, soak them in NaOH (0.1 M) for 24 h, and then rinse them with cold deionized water until the solution is neutral. Decalcification was performed in 0.5 mol/L ethylenediaminetetraacetic acid disodium salt (EDTA-2Na) (pH 7.4, 1:10, *w*/*v*) with continuous stirring for 48 h to remove non-collagenous compounds, and the solution was changed every 8 h.

The residue was stirred in 15 volumes of acetic acid (0.5 M) containing 0.1% (1:6, *w*/*v*) pepsin for 24 h. The supernatant was collected after centrifugation at 10,000× *g* and 4 °C for 30 min. The pepsin was inactivated by adding sodium hydroxide and the pH value was adjusted to 5. The rest of the residue was re-extracted with acetic acid containing pepsin by following the same procedures. The supernatants were combined twice, and NaCl (2 M) was then added for salting out. Collagen precipitates were collected by centrifugation at 10,000× *g* for 30 min, dissolved in acetic acid (0.5 m), and then dialyzed with distilled water. A freeze dryer was used to freeze-dry the dialysate (7420070, Labconco, Kansas City, MO, USA) to obtain pepsin-solubilized collagen from Tilapia skull, NTCII.

### 2.3. Sodium Dodecyl Sulfate-Polyacrylamide Gel Electrophoresis (SDS-PAGE)

SDS-PAGE analysis was performed using the method of Laemmli [16]. Dissolve the freeze-dried samples with 5% SDS, treated in a water bath at 80 °C for 1 h, as well as, centrifuged at 10,000× *g* for 5min at a normal atmospheric temperature. Afterward, 30 μL supernatant and 10 μL protein loading buffer were mixed and maintained in a 95 °C water bath for 5 min. Then 10 μL treated samples wasloaded onto polyacrylamide gel containing 8% separating gels and 5% accumulated gels and electrophorsed at 110 V constant voltage in the DYY-2c electrophoresis device (Beijing Liuyi Biotechnology Co., Ltd, Beijing, China). The gel was stained with Coomassie blue R-250 for 30 min. Finally, the gel was destained for 24 h and was visualized in a Biospectrum-500 Gel Imaging Analyzer (Cambridge, UK).

### 2.4. Amino Acid Composition

The lyophilized NTCII was hydrolyzed with HCl(6M) at 110 °C for 24 h. Adjust pH to 7. Dilute the filtrate to 20 mL with ionic water. Remove 400 μL of the solution and adjust the volume was to 2 L with HCl (0.02 M); in addition, take 1ml of the solution and pass through amembrane filter (0.45 μM), then use an amino acid analyzer (Hitachi l-8900, Japan) for quantitative analysis.

### 2.5. Fourier Transform Infrared (FTIR) Analysis

The lyophilized NTCII was mixed with the dried pure potassium bromide (KBr) (1:100, *w*/*w*) and pressed into flakes under dry conditions and subjected to FTIR analysis using an FT/IR-410 spectrometer (TG209 F1, Netzsch, Germany) 4000–400 cm^−1^ (mid-IR region) at 25 °C.

### 2.6. Thermal Denaturation Temperature

The thermal denaturation temperature was measured by differential scanning calorimetry (DSC). NTCII was weighed and placed in a crucible. In order to obtain DSC spectra, NTCII was scanned by a differential scanning calorimeter at 20–150 °C.

### 2.7. Polysaccharide Content

Use a glycoprotein/carbohydrate evaluation kit (Thermo Scientific Catalog #23260, Shanghai, China) to determine the percentage of glycosylation. In brief, NTCII was diluted to 0.25 mg/mL and 2.5 mg/mL with glycoprotein analysis buffer. Samples and standards were tested in triplicate in a microtiter plate at 50 μL/well. Fill the blank sample wellwith glycoprotein analysis buffer. Dispose of the sample in accordance with the manufacturer’s instructions.

### 2.8. Markings for NTCII and Detection Marker Results

NTCII was dissolved in Na_2_CO_3_-NaHCO_3_ buffer solution, to make a 1 mg/mL solution. Subsequently, FITC was added while stirring under light-proof conditions, and the ratio of the amount of FITC to NTCII was about 1:10. The sample was placed in a low-temperature environment at 4 °C and stirred for 24 h. The solution of FITC-NTCII was added into a dialysis bag with a cut-off molecular weight of 500, dialyzed for 3–5 days and freeze-dried when protected avoidance of light. The dried powder was placed in a desiccator for storage away form light.

The above-labeled samples were diluted to 5-fold and 10-fold, respectively for SDS-PAGE analysis. The entire process was performed in the dark. After the gel was run, it was placed in a gel imager and illuminated with green fluorescence.

### 2.9. CD8^+^ T Cells Culture

The cells were cultured with RPMI-1640 (10% fetal bovine serum and 1% penicillin-streptomycin) in an incubator (humidified at 5% CO_2_ and 37 °C) for proliferation study. Cells in good growth condition and the log phase were subjected to follow-up experiments.

### 2.10. Cytotoxicity Assay

The CCK-8 kit was used to detect the cytotoxicity of NTCII to CD8^+^ T cells. Cells in the logarithmic growth stage were cultured in 96-wellPLATES, and about 4000 cells per well were cultured in a cell incubator for 24 h. Different concentrations (1, 10, 25, 50, and 100 μg/mL) of NTCII were added to CD8^+^ T cells. Samples with-out collagen served as the control group. The cells were cultured in an incubator for 48 h, then 10 μL of CCK-8 solution was added, and the cells were cultured for 4 h. The absorbance was measured with a microplate reader (SH-1000, Hitachi, Japan) at 450 nm.

### 2.11. Effect on the Cytokine Content of CD8^+^ T Cells

The ELISA kit method was used to determine the relevant cytokines of cell supernatant (a double antibody sandwich assay). The cells in the logarithmic growth phase were cultured in 6-well plates for 12 h in a cell culture incubator. Different concentrations (1, 10, 25, 50, and 100 μg/mL) of NTCII were added to CD8^+^ T cells. No collagen was added to the control group. After 24 h, 1 μg/mL lipopolysaccharide (LPS) was added for treatment and the cells were placed in an incubator for up to 48 h. The supernatant was collected after centrifugation (2000× *g*, 30 min). The ELISA detection kit method was used to detect the Fas/Apo-1 (IL-1 β), (IL-6), and TNF-α in the microplate reader.

### 2.12. Quantitative Real-Time PCR (qPCR)

Total RNA was isolated from CD8^+^ T cells with TRIzol reagent (Invitrogen, Waltham, MA, USA). After treatment with DNAse, RNA was reverse transcribed to single-strand cDNA using a cDNA first-strand synthesis kit. qRT-PCR was performed using SYBR Green Premix Ex Taq on an ABI 7500 real-time PCR Detection System (Applied Biosystems, Shanghai, China) (Table 1).

The primer pairs targeted FAS, caspase-3, and caspase-8 (Table 2). The relative expression of target genes was measured by the CT value of the internal control group and each experimental group (2^−^^ΔΔCT^) [17].

### 2.13. Binding Status of FITC-NTCII to CD8^+^ T Surface Antigen Receptors

The cell concentration was adjusted to 1 × 10^6^ cells/mL with RPMI1640 culture medium; then 200 μL was added to a 96-well plate and FITC-NTCII was added to a concentration of 3 μg/mL, 5 μg/mL, 10 μg/mL; or 50 μg/mL, and incubated for 24 h at 37 °C in a 5% CO_2_ incubator in the dark. The supernatant was discarded, washed three times with PBS and photographed under a fluorescent inverted microscope.

### 2.14. Arthritis Study

The rats (4–6 weeks) were purchased from GemPharmatech Co., Ltd., Shanghai, China and housed for 2 weeks to acclimatize the environment under light and dark cycle with free access to water and a normal diet. The animal experiments were conducted as per the standard procedure of Shanghai Ocean University (Approval Number: C57BL6N).

After 14 days of adaptive feeding, the animals were randomly divided into four groups containing 10 rats: the control group, blank group, TCII-1 group, and TCII-2 group. Control group: animals receiving no treatment; blank group (the inflammatory group): arthritis-induced animals were given 0.2 mL of 0.05 mol/L acetic acid solution for 4 weeks; TCII-1 group: arthritis induced animals were given 0.2 mL of 1 mg/mL NTCII solution for 4 weeks and NTCII-2 group: arthritis induced animals were given 0.2 mL of 3 mg/mL NTCII solution for 4 weeks. Arthritis was induced in rats by the subcutaneous injection of 0.1 mL CFA (complete Freund’s adjuvant) into the left hind toe for 2 weeks. Each group used a gastric feeder for oral administration once a day and continued oral administration until the material was taken.

### 2.15. Preparation of Pathological Sections of Synovium of Rat Joints

Four weeks after oral administration, the rats were taken blood and sacrificed by neck dislocation. Three rats were randomly selected form the blank, control, NTCII-1, and NTCII-2 group, and handled in a sterile environment. After the removal of the ankle joint, the rats were fixed in a supine position on an ultra-clean workbench, and the surgical field was routinely disinfected with iodine and alcohol. The skin was excised longitudinally along the middle of the knee joint, about 0.5 × 0.5 cm, the patella was cut about 0.3–0.4 cm along the upper edge using toothed forceps, and then the synovial tissue was completely peeled off and immediately fixed in formalin solution. After decalcification, the tissue was washed with running water and dehydrated with conventional gradient ethanol series, embedded in paraffin, sectioned (4 μm thickness, Lycra microtome), stained with HE and observed under a high-magnification stereo microscope [2].

### 2.16. Statistical Analysis

Statistical analyses were performed using GraphPrism9. Shapiro-Wilk test (for sample size < 50) was used to evaluate the normality of the quantitative variables. Differences between the groups based on one-way ANOVA were calculated by Prism 9statistical package. The probability value of <0.05 was considered significant. All data were reported as mean ± SD. All experiments were performed in triplicate (*n* = 3).

## 3. Results and Discussion

### 3.1. SDS–PAGE Protein Pattern of Collagen

**Figure 1:**
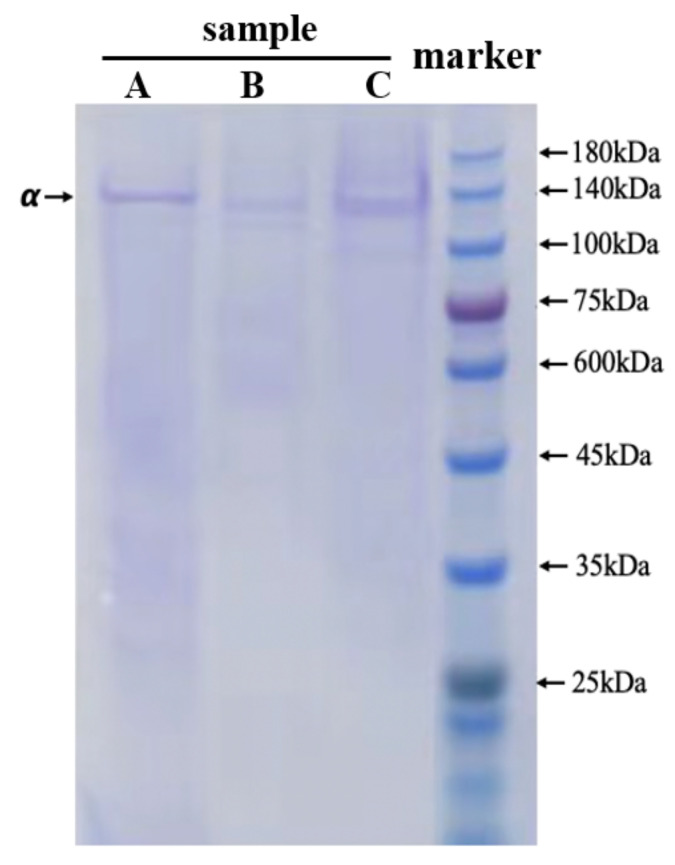
SDS-PAGE patterns of NTCII and standard protein marker. A—Pepsin solubilized collagen from chicken breast cartilage (presented by Shanghai Fisheries Research Institute), B—Pepsin-solubilized collagen from Andriasdavidianus cartilage (presented by Shanghai Fisheries Research Institute), C—Pepsin-solubilized collagen from Tilapia skull, and 4—Marker.

The electrophoretic protein pattern of NTCII was presented in Figure 1. The collagen samples were composed of three identical α1-chains with no high molecular weight bands such as beta and gamma, and the molecular weight of the α-chain was approximately 130 kDa. The present result was in agreement with the collagen extracted from the cartilage of the silvertip shark [15] and confirmed that the extracted collagen was in the type II category and was complete. It is already known that type II collagen, which is mainly distributed in cartilage, is composed of three identical α1-chains [16,17]. The molecular size and amino acid composition of collagen are closely related to its physicochemical and functional properties [18].

**Figure 2:**
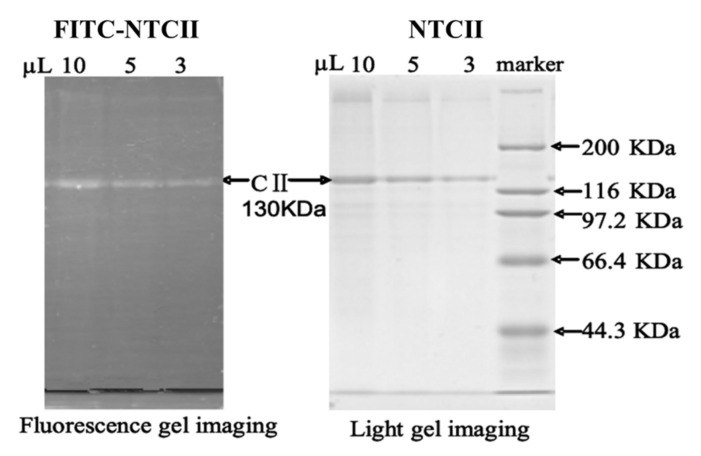
The result of fluorescent labeling by SDS-PAGE.

The electrophoretic protein pattern of FITC-NTCII is presented in Figure 2. A bright band in each lane can be seen in the fluorescence photogram, which was stained with Coomassie Brilliant Blue and photographed under white light conditions, revealing a band with a molecular weight of 130 KDa in each lane. This result corresponds to the bright band photographed under fluorescence conditions, thus indicating that FITC successfully combined with NTCII and that these bright bands are the result of FITC-NTCII under green excitation light. At sample loading levels of 10 μL, 5 μL and 3 μL for electrophoresis, increasing weakness in the fluorescence intensity of the bands could be seen, but even at lower loading levels, the fluorescence intensity remained strong, indicating that FITC was firmly bound to NTCII at a high concentration.

### 3.2. Amino Acid Composition

The amino acid composition (expressed as residues/1000 residues) of the collagens from the Nile tilapia skull are shown in Table 3. Amino acid analysis indicated that NTCII consisted of 315/1000 glycine residues, 72/1000 hydroxyproline residues and 108/1000 proline residues. Glycine was the most plentiful amino acid in NTCII, accounting for 31.46% of the total amino acids. In addition to glycine, the contents of glutamic, proline, and alanine in NTCII were lower than those in other collagen samples. On the other hand, histidine (9 residues/1000 residues) and methionine (4.84 residues/1000 residues) were present at a low amount in NTCII, and no tryptophan residues were found. The imino acids (proline and hydroxyproline) content of NTCII was 141.11/1000 residues, which was similar to that of collagens in Pacific cod skin (157/1000 residues) [19], and higher than that in Mahi-mahi skin (126.4/1000 residues) [20]. The collagen molecular region rich in hydroxyproline and proline participates in the formation of hydrogen bond stability and limits the conformation of a polypeptide chain, thus helping to strengthen the triple helix [21,22]. The content of NTCII imino acids was higher than in the other types of collagens. Collagen has been demonstrated to be an alternative source of antioxidant peptides and antihypertensive peptides due to the strong inhibitory activity of hydrophobic amino acids (such as alanine, threonine, valine, proline, isoleucine, and phenylalanine) at the C-terminus [23]. Notably, the content of medicinal amino acids in NTCII collagen was high.

### 3.3. Fourier Transform Infrared (FTIR) Spectra

**Figure 3:**
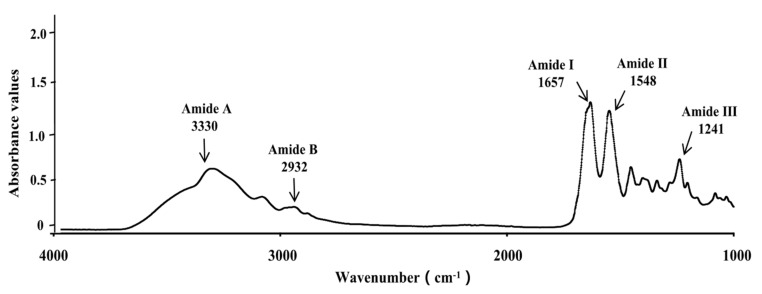
FTIR spectra of NTCII.

The FTIR spectra of NTCII displayed the characteristic peaks of amide I, II, III, A, and B (Figure 3). The main peaks of NTCII spectra were similar to those of other fish collagen [24]. Similar FTIR spectra were observed for NTCII and other fish collagens. The characteristic absorption peak of amide occurs in the wavenumber range of 3400–3440 cm^−1^, which is usually related to the N-H stretching vibration. When the NH group of the peptide participates in the hydrogen bond, the characteristic peak position will move to a lower frequency [27]. The absorption peaks of NTCII were found at 3330 cm^−1^. However, in this current investigation of the NTCII samples, the amide A band signal moved to a lower frequency at 3300 cm^−1^. This may be due to the involvement of hydrogen bonds in the NH groups of collagens or the combination of H-bond and COO [28]. The amide B band of NTCII was observed at the wavenumber of 2932 cm^−1^, which was related to the asymmetric stretching of CH_2_ and NH^3+^ [21]. The sharp amide I band of NTCII was observed at 1657 cm^−1^, which was similar to other studies [29]. It is mainly associated with the C=O stretching vibration along the polypeptide backbone [30] and is used to analyze the secondary structure of proteins [15]. The amide II of NTCII appeared at 1548–1553 cm^−1^, resulting from the coupling of the N-H bending vibration and CN Tensile vibration [31]. The absorption peak of amide III was located at 1236–1241 cm^−1^, which was NTCII isolated collagen. The amide III peak reflected intermolecular interactions in collagen, including N-C-N stretching and the N-H deformation of amide bonds, which is also related to the triple-helical structure [31]. The absorption ratio between the amide 3 band and 1430 cm^−1^ was about 1, which was indicated the triple helix structure of collagen [32]. The ratios of NTCII was 1.15. This confirmed that the triple-helical structure of the collagens from skull of Nile tilapia was still intact, and most of the intermolecular structure remained intact.

### 3.4. Thermal Denaturation Temperatureand Polysaccharide Content

**Figure 4:**
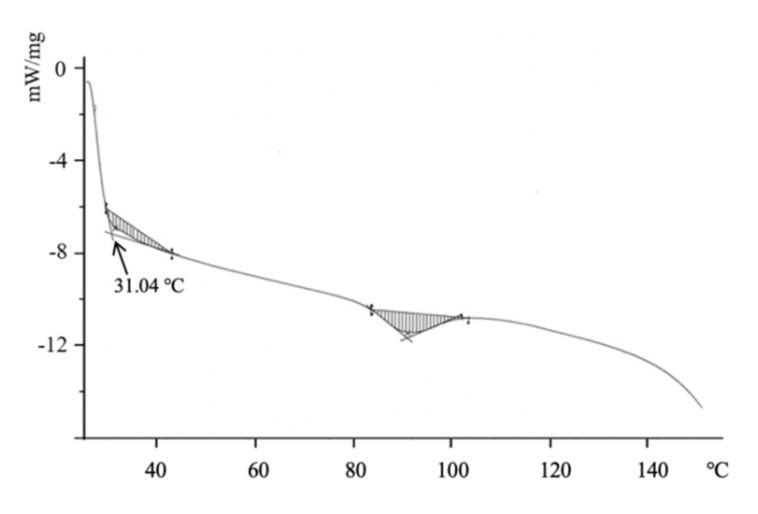
DSC curve of NTCII.

The thermal denaturation temperature of type II collagen was determined by DSC. At the thermal denaturation temperature, the triple helix structure of type II collagen unfurled into random coils, which caused collagen to lose its unique properties. The DSC spectra of the NTCII are shown in Figure 4. The thermal denaturation temperature (Td) of NTCII was 31.04 °C and the glass transition temperature of NTCII was 91.09 °C. Usually, the thermal denaturation temperature of collagen from terrestrial mammals is higher than that of fish collagen, which also limits the application of fish collagen. The thermal stability of collagen is generally associated with the content of imino acids, glycosylation percentages, and the triple helix structure. The pyrrole ring in sub-imino acids and glycosylation helped to maintain the stability of the collagen triple helix structure. In this research, the Td of NTCII was higher than that of cold-water fish collagen [19,20].

The range of glycosylation percentages in NTCII was 5.6–19.0%. Jeevithan et al. showed that the glycosylation of type II collagen in silver shark cartilage was 5.36–32.3% [15]. Ningchao et al. showed that phytoestrogens protect joints in collagen-induced arthritis by increasing IgG glycosylation and reducing osteoclast activation [33]. Thompson et al. reported the suppression of collagen-induced arthritis by the oral administration of type II collagen [9].

### 3.5. Effect of NTCII on the Growth of CD8^+^ T Cells

**Figure 5:**
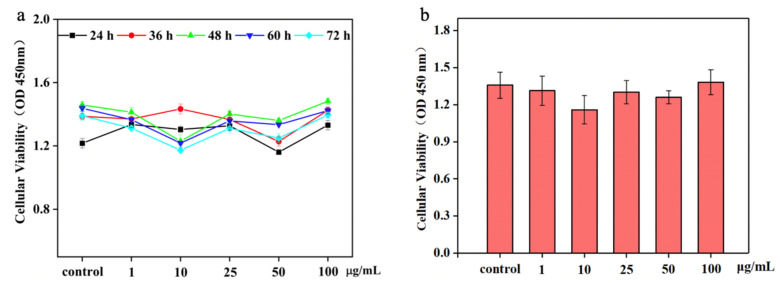
Effects of cell proliferation of varying concentrations of NTCII. (**a**) Cell survival at different time points. (**b**) Cell proliferation at 48 h.

The viability and proliferation of CD8^+^ T cells are shown in Figure 5. In general, a slight variation in the absorbance values of all experimental groups was observed, which confirmed that collagen has a certain inhibitory effect on CD8^+^ T cells. The number of CD8^+^ T cells was decreased after treatment with NTCII concentrations of 1 μg/mL, 10 μg/mL, 25 μg/mL and 50 μg/mL. As the incubation time increased, the rate of apoptosis in the group with added NTCII was accelerated compared to the control group. The above results concluded that the highest inhibitory effect on CD8^+^ T cells was found in the 10 μg/mL NTCII-treated group, and the inhibition rate was 14.67%, but there was no significant difference.

A previous study reported that the induction of apoptosis in T lymphocytes was regulated by type II collagens through glycoprotein binding with tumor necrosis factor receptor (TNFRSF6) in the cell membrane, which activates the caspase cascade pathway [34].

### 3.6. Inflammatory Cytokine Content in CD8^+^ T Cells

**Figure 6:**
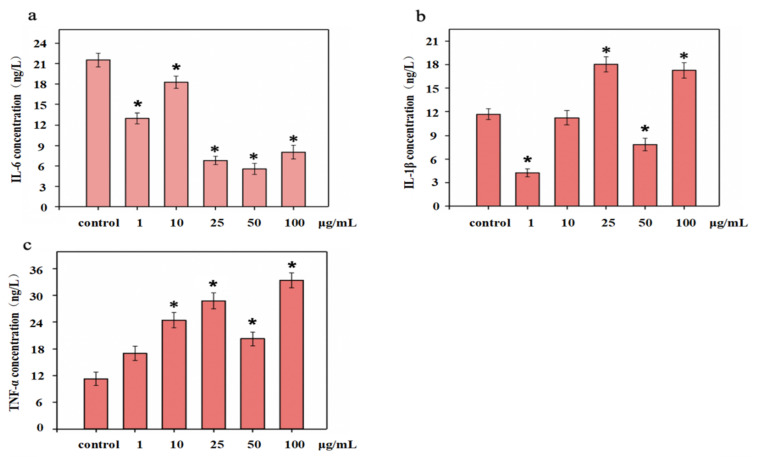
Levels of cytokines in CD8^+^ T cells treated with varying concentrations of NTCII before LPS stimulation ((**a**) IL-6, (**b**) IL-1β, (**c**) TNF-α). (Shapiro-Wilk value > 0.05, using one-way ANOVA and Dunnette for post hoc tests.) An asterisk indicates that there is a significant difference compared with the control group (* *p* < 0.05).

Cytokines are smaller proteins released from immune cells under specific conditions triggered by antigens. They were mainly involved in cell signal transduction pathways that transmit signals to other cells to activate the defense system. Figure 6 showed the cytokine levels in CD8 ^+^ T cells treated with different concentrations of NTCII before LPS stimulation. The effects on IL-6 were shown in Figure 6a. The expression of IL-6 in the control was 21.53 ng/L, and when the concentration was 1 μg/m, 10 μg/m, 25 μg/mL, 50 μg/mL and 100 μg/mL, the concentration of IL-6 was 12.99 ng/L, 18.27 ng/L, 6.81 ng/L, 5.56 ng/L and 8.02 ng/L, respectively, compared with the control group, NTCII significantly accelerated the gene expression of IL-6 (*p* < 0.05).

**Figure 7:**
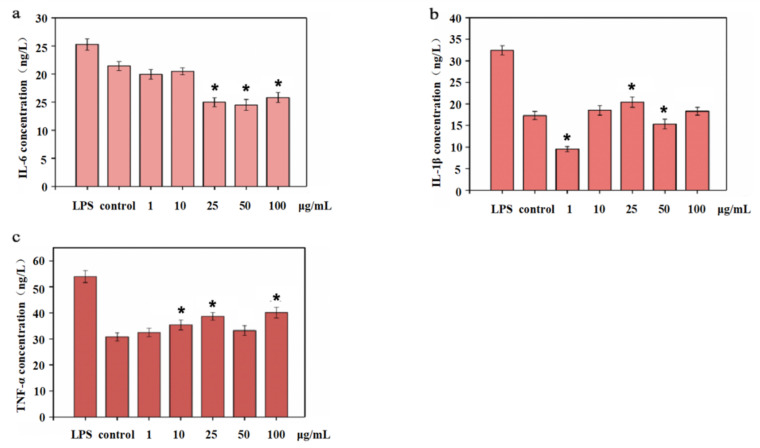
Level of cytokines in CD8^+^ T cells treated with varying concentrations of NTCII after LPS stimulation ((**a**) IL-6, (**b**) IL-1β, (**c**) TNF-α). (Shapiro-Wilk value >0.05, using one-way ANOVA and Dunntte for post hoc tests.) An asterisk indicates that there is a significant difference compared with the control group (* *p* < 0.05).

IL-1β can stimulate the production of IL-6; therefore, a decrease of IL-1β secretion also inhibits the secretion of IL-6. Collagen can regulate the secretion of cytokines in lymphocytes, thereby affecting the immune response of cells. As shown in Figure 7b, the secretion of IL-1β in the control group was 11.71 ng/L. For NTCII addition of1 μg/mL and 50 μg/mL, IL-1β expression was 4.24 ng/L and 7.85 ng/L, respectively. Compared with the control group, the expression of IL-1β was dramatically reduced.

NTCII increased the secretion of TNF-α in each concentration group. As shown in Figure 7c, when NTCII concentration reached 25 μg/mL and 100 μg/mL, the secretion of TNF-α was 28.73 ng/L and 33.36 ng/L, significantly higher than that of the control group (*p* < 0.05).

### 3.7. Influence of Apoptosis Factors on CD8^+^ T Cells

**Figure 8:**
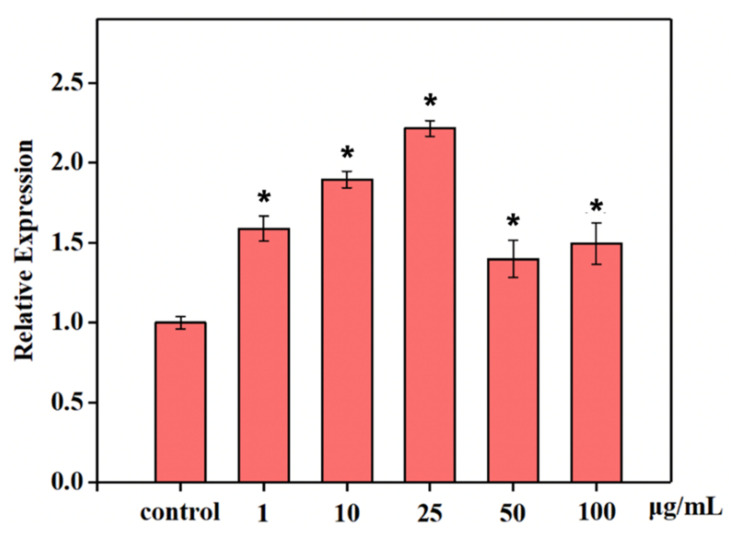
Content of cytokines Fas/Apo-1 in CD8^+^ T cells treated with varying concentrations of NTCII. (ShapiroWilk value > 0.05, using one-way ANOVA and Dunnette for post hoc tests.) An asterisk indicates that there is a significant difference compared with the control group (* *p* < 0.05).

Fas/Apo-1 is a cell surface protein that can regulate cell apoptosis. Fas/Apo-1 can up-regulate the apoptosis in different living cells by stimulating apoptotic signals. As shown in Figure 8, the levels of Fas/APO-1 were significantly higher in CD8^+^ T cells cultured with NTCII than in the control group, indicating that collagen can down-regulate the level of the cellular immune response. With the increase in collagen concentration, NTCII promoted the secretion of Fas/Apo-1. Compared with the control group, 1, 10, 25, 50 and 100 μg/mL of NTCII treatment resulted in higher Fas/Apo-1 secretion levels in CD8^+^ T cells. Not only Fas/Apo-1, but the cytotoxic effect of collagen on CD8^+^ T cells could also regulate by other factors and signaling pathways. In the present study, NTCII could promote apoptosis in CD8^+^ T cells, but the increase varied with different concentrations of collagen. The main reason may be related to the degree of collagen hydroxylation. In the previous study, it has been reported that different molecular weight collagen polypeptides increased the level of apoptosis and cytotoxicity 6T-CEM cell, which may be regulated by their degree of glycosylation [34].

### 3.8. Expression of Apoptosis-Regulating Genes in CD8^+^ T Cells

**Figure 9:**
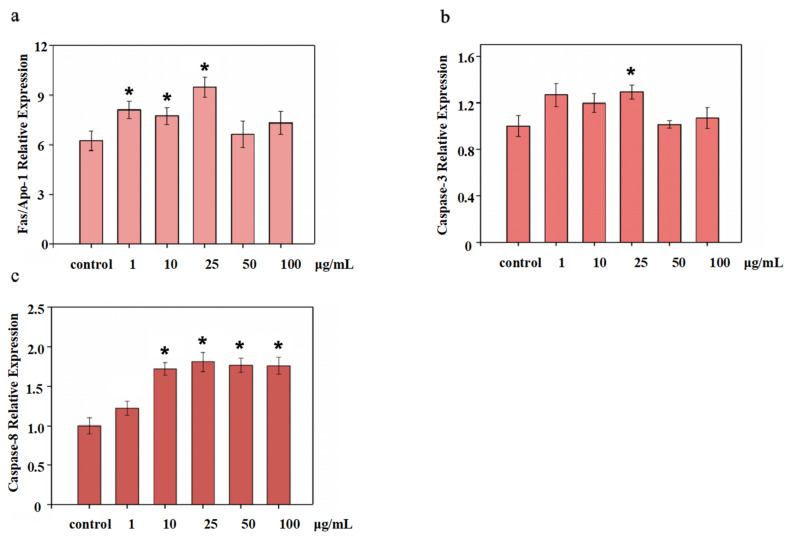
Gene expression of CD8^+^ T cells by real-time PCR treated with varying concentrations of NTCII. (**a**) Fas/Apo-1; (**b**) Caspase-3; (**c**) Caspase-8. (Shapiro-Wilk value > 0.05, using one-way ANOVA and Dunnette for post hoc tests.) An asterisk indicates that there is a significant difference compared with the control group (* *p* < 0.05).

Asparaginase (caspase) triggers antigen stimulation in the CASPASE cascade reaction pathway, which activates the immune system for apoptosis. In this research, compared to the control group, NTCII was shown to stimulate cells to secrete high levels of caspase-3 and caspase-8. As shown in Figure 9a, when the concentration of NTCII was 25 μg/mL, the expression of Fas/Apo-1 reached 9.48 μg/L, which was significantly higher than that of the control. As shown in Figure 9b, the expression of the caspase-3 gene was significantly higher when NTCII was added at a concentration of 25 μg/mL. As shown in Figure 9c, compared with the control group, the expression of the caspase-8 gene was up-regulated in the NTCII group (*p* < 0.05). When the concentration of NTCII increased from 1 μg/mL to 10 μg/mL, the NTCII groups resulted in a significant increase in the expression of the caspase-8 gene in (*p* < 0.05) compared to the control group. However, as the concentration increases further, caspase-8 did not change much, which shows that it has no concentration dependence on NTCII.

The NTCII could alter the levels of caspase-3, FAS, and caspase-8. The degree of change is related to molecular size, amino acid composition, and molecular structure characteristics. From this experimental result, type II collagen can promote CD8^+^ T cells apoptosis by activating the Fas signal channel. It promotes the expression of intracellular cytokines, which triggers the down-regulation of immune response by gene regulation. Normally, by activating caspase-3, caspase-9, and caspase-8, the CASPASE cascade reaction is triggered, causing exogenous apoptosis [35]. Under normal circumstances, NTCII can stimulate the expression of apoptotic cytokine and mRNA in CD8^+^ T cells.

The above in vitro results proved that the collagen II treatment downregulated the proliferation of the immune cells, thereby reducing the sensitization risk and hypersensitivity. Our previous also study confirmed the low antigenicity of marine biological collagen [10]. Based on the sensitization reaction risk in collagens type II studies, the acute systemic toxicity after injection of shark type II collagen was measured to test its practical applicability. There was no difference in death rate between the control group and the collagen treatment group, and in general, there was no significant difference in behavioral (motor) activity, abdominal irritation, respiratory diseases, eyelid prolapse, and normal behavior [10]. In contrast, earlier reports claimed that the immunoglobulin E (IgE) level was increased in Japanese patients who had fish allergy by about 50% for mackerel collagen and 44% for mackerel parvalbumin [36]. In addition, the study highlighted that fish collagen accelerated IgE cross-linking on mast cells in-vitro. Bobadilla et al. studied the sensitivity of collagen type V by trans-vivo delayed-type hypersensitivity reaction and found that collagen type V sensitization was related to bronchiolitis obliterans syndrome, which leads to gastroesophageal reflux disease [37]. Similarly, the sensitization risk of collagen type II by humoral or cellular mechanisms was previously well studied by several researchers [38,39,40]. These findings revealed that fish collagen may perpetuate a low-level sensitization risk (such as common panallergens), especially to people with fish allergies.

### 3.9. Analysis of Specific Binding of FITC-NTCII to CD8^+^ T Cells Surface Receptors

**Figure 10:**
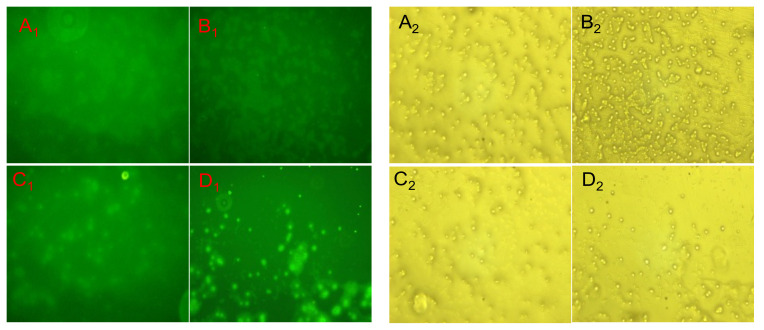
The fluorescent and white light images of FITC-NTCII P combined with CD8^+^ T cells at different concentrations. (**A_1_**,**B_1_**,**C_1_**,**D_1_**) were the results of CD8^+^ T cells surface antigen receptor binding fluorescence photographed after 5 days of co-culture with CD8^+^ T cells at 3 μg/mL, 5 μg/mL, 10 μg/mL and 50 μg/mL of FITC-NTCII respectively; (**A_2_**,**B_2_**,**C_2_**,**D_2_**) were the results of their corresponding white light conditions.

The corresponding images of FITC-TC II at 3 μg/mL, 5 μg/mL, 10 μg/mL and 50 μg/mL in fluorescence and white light are shown in Figure 10.

As can be seen from Figure 10, FITC-NTCII binds to specific receptors on the surface of CD8^+^ T cells after co-culture with CD8^+^ T cells protected from light. As the concentration of FITC-NTCII increased, the results of the images taken under fluorescent conditions became clearer; thus, the fluorescence intensity was proportional to the concentration of FITC-NTCII. In other words, the higher the concentration of FITC-NTCII, the more FITC-NTCII bound to CD8^+^ T cells; therefore, the stronger the fluorescence intensity seen under fluorescence microscopy. This also demonstrates that FITC-NTCII binds successfully to the surface receptors of CD8^+^ T cells.

### 3.10. Fish Type II Collagens Promote the Repair of the Joint Cartilage

**Figure 11:**
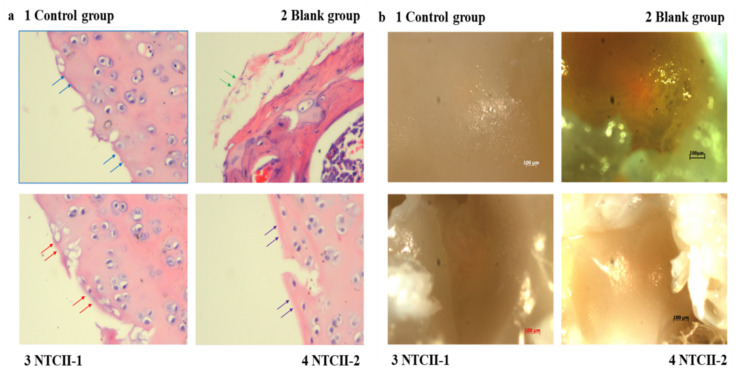
(**a**) The effect of collagen on the histopathology (HE staining) of the rat ankle joint tissues. (**b**) The high magnification stereo microscope of rat ankle surface. (Blue arrows show smooth surface with dense protein layer; green arrows show damage; red arrows show recovery; and purple arrows show fully recovered tissues. Control group: animals treated without any treatment, blank group: arthritis-induced animals were treated with 0.2 mL of 0.05 mol/L acetic acid solution for 4 weeks; NTCII-1 group: arthritis-induced animals were treated with 0.2 mL of 1 mg/mL NTCII solution for 4 weeks; and NTCII-2 group: arthritis-induced animals were treated with 0.2 mL of 3 mg/mL NTCII solution for 4 weeks.).

After 14 days of oral treatment, the rats were sacrificed and their ankle joints were sectioned. Figure 11a shows that the HE-stained surface of the articular cartilage in the blank group was smooth, without damage, whereas the HE-stained surface in the articular cartilage in the control group was uneven, was being repaired in the NTCII-1group and, finally, the HE-stained surface of the articular cartilage in the NTCII-2 group was smooth (fully recovered as the blank group).

In the blank group, the hyaline chondrocytes were arranged regularly and neatly, with clear levels. In the control group, the NTCII-1 group, and the NTCII-2 group, the joint area was damaged and the cells were small and immature, densely arranged, irregular and chondrocyte-like cells. Most of the articular cartilage at the junction of normal tissue and repaired tissue was dead, which is manifested by the sparse number of cells in the junction area. The present result showed that after the ankle joint of the rats in the NTCII-2 group recovered, the joint surface was as smooth as that of the blank group, indicating that the effect of NTCII-2 was better than that of the NTCII-1 group.

During the decalcification process, the articular cartilage was observed under a high magnification stereo microscope. Figure 11b shows the surface of the rat ankle joint treated with type II collagen. Similar to the HE-stained images, the surface of the ankle joint of the control group was uneven and the color was yellowish. The surface of the ankle joint of the blank group, the NTCII-2 group, and the NTCII-1 group was smooth, indicating that the effects of NTCII-2 and NTCII-1 were consistent, and the ankle joint surface was repaired. The present study confirmed the effect of NTCII-2 on the ankle joint was similar to NTCII-1, which proved that marine collagen could promote the recovery of joint synovium in rats.

Similar to the present study, the effect of type II collagen on a synovial membrane and ankle-joint health was studied by many researchers [41,42]. In addition, the actual mechanism of type II collagen in the articular cartilage health protection is explained by numerous hypotheses [42]. It was stated that the oral tolerance of collagen type II was attributed to the modulation of the cellular inflammatory pathways [43].

## 4. Conclusions

NTCII had a unique super-helical twist in three peptide chains and many component polysaccharides, and is composed of monosaccharides that belong to marine fibrous glycoprotein. The polysaccharide content of NTCII was 5.36–32.3%. NTCII has high thermal stability; the highest thermal denaturation temperature is 30.43 °C, and can down-regulate inflammatory factors. The present research demonstrates that the NTCII binding to CD8^+^ T cells can trigger rigidity. The results showed that the levels of apoptotic mediators, such as the gene expression of Fas/Apo-1 level, caspase-3, and caspase-8 in CD8^+^ T cells were not significantly altered in vitro even at very low concentrations of collagen. This observation disclosed that rigidity did not relate to an increase of inflammatory factors in the CD8^+^ T cells by NTCII. It is suggested that the rigid or apoptotic T cells were supposed to control RA because the percentage of T cell TIGIT (immunoreceptor with Ig and immunoreceptor tyrosine-based inhibitory domains) expression in the peripheral blood CD8^+^ T cells of RA patients was positively correlated with rheumatoid factors. NTCII also supports the treatment of the RA model in vivo, which can repair the surface of the ankle cartilage from damaged to smooth. The oral administration of 3 mg/kg NTCII in the RA rat model could effectively repair the damaged ankle cartilage. The levels of TNF-α, IL-6, and IL-10 in plasma were decreased by 1 mg/kg and 3 mg/kg doses of NTCII. The effect of NTCII on rigidizing CD8^+^ T cells was further supported by the orally administered animal model.

This study clearly demonstrates that there is a close relationship between the molecular properties and characteristics of induced CD8^+^ T cell apoptosis in vitro and in repairing ankle joint injury in an in vivo RA model in NTCII, a functional material from marine fibrous glycoprotein. These data also demonstrated that the effect of NTCII in rigid T cells and oral tolerance did not contribute to any inflammation and its determinant with Fas/Apo-1 and histomorphology. NTCII could be used as an alternative source of mammalian collagen for further RA applications. This study revealed the immunological and anti-arthritic effects of fibrillar collagen and expands the range of choices for collagen applications.

## Figures and Tables

**Table 1:** Quantitative RT-PCR reaction solution and program set-up.

PCR Program	Sample (μL)	Temperature (°C)	Time (s)	Total Cycles
2× Talent qPCR Premix	10	95	180	35
Primer (F + R)	2	95	10	
cDNA	1	58	30	
RNase-Free ddH_2_O	6.6	72	30	
50× ROX Reference Dye	0.4			

**Table 2:** Primer sequences were used in this study.

Primer Name	Primer Sequence (5′ to 3′)
Fas/APO-1-F	GGACCCAGAATACCAAGTGCAG
Fas/APO-1-R	GTTGCTGGTGAGTGTGCATTCC
Caspase3-F	GGAAGCGAATCAATGGACTCTGG
Caspase3-R	GCATCGACATCTGTACCAGACC
Caspase8-F	AGAAGAGGGTCATCCTGGGAGA
Caspase8-R	TCAGGACTTCCTTCAAGGCTGC
β-Actin-F	AGCGAGCATCCCCCAAAGTT
β-Actin-R	GGGCACGAAGGCTCATCATT

**Table 3:** Amino acid compositions of NTCII and other collagen samples (residues/1000 residues). NTCII-Pepsin-solubilized collagen from Nile tilapia skull.

Amino Acids	NTCII	Tuna Spine [24]	Pacific Cod Skin [19]	Pike Scale [25]	Seabass Scale [26]
Asp	55	44	55	41	44
Thr	28	30	23	22	24
Ser	41	40	61	28	28
Glu	83	78	81	67	71
Gly	315	332	337	363	327
Ala	117	110	107	122	133
Val	21	34	21	20	22
Met	10	0.0	11	16	15
Ile	22	21	13	10	11
Leu	23	28	25	20	21
Tyr	3	0.0	4	2	5
Phe	23	23	14	12	14
Lys	22	31	27	27	27
His	9	3	11	5	7
Arg	54	51	51	54	52
Pro	108	99	93	124	108
Hyp	72	70	64	66	85

## Data Availability

All data included in this study are available upon by contact with the corresponding author.
